# EXPLORING MOBILE PAIN APPLICATIONS WITH DIVERSE ELDERS

**DOI:** 10.1093/geroni/igae098.1711

**Published:** 2024-12-31

**Authors:** Mary Milidonis, Hayley Rupp, Patrick McGinty, Karen Kopera-Frye

**Affiliations:** Cleveland State University, Cleveland, Ohio, United States; Cleveland State University, Cleveland, Ohio, United States; Cleveland State University, Cleveland, Ohio, United States; New Mexico State University, Las Cruces, New Mexico, United States

## Abstract

Mobile pain applications (apps) may improve health communication. Mobile health apps do not always consider the needs of diverse older adults. This qualitative project examines perceptions of smartphone pain apps between African American and Caucasian older adults. The sample consisted of 19 older adults (mean age = 68 years), with majority female (88%), predominantly Caucasian (76%), African American (24%) and majority some college education (70%). Participants were randomly assigned to evaluate 2 of 5 free pain apps. Respondents answered questions about pain apps using the Mobile Application Rating Scale (MARS). The MARS assesses descriptive and technical aspects of an eHealth apps using five categories including four quality scales: engagement, functionality, aesthetics, and information and one subjective quality scale. Participants experienced persistent pain from 3 months to 12 years. Participants self-reported an average of 2.6 comorbidities on the Functional Comorbidity Scale. Important characteristics identified by both groups of older adults as critical for useable app included: Clear instructions and good navigation. The African American group differed on what would deter use of the app which included fear of use, uncertainty about the app and preferred use of personal physician. The Caucasian group themes for what deterred app use included complexity and lack of engagement. Both groups identified the important features were tracking pain and treatment ideas. These are important considerations in designing health technology for use with diverse older adults who need recurrent pain self-management.

